# Microbe-mediated nanotechnology for heavy metal detoxification and crop resilience: mechanisms, advances, and future prospects

**DOI:** 10.3389/fmicb.2026.1896768

**Published:** 2026-07-29

**Authors:** Farwa Basit, Hao Wang, Vishwa Deepak, Jing Nie, Qian Wang, Saad Sulieman, Ali El-Keblawy, Zein El-Abedin A. Mohamed, Mohamed S. Sheteiwy

**Affiliations:** 1 Department of Biology, College of Science and Technology, Wenzhou-Kean University, Wenzhou, China; 2 Department of Integrative Agriculture, College of Agriculture and Veterinary Medicine, United Arab Emirates University, Al Ain, United Arab Emirates; 3 Department of Applied Biology, College of Sciences, University of Sharjah, Sharjah, United Arab Emirates; 4 Department of Agricultural Botany, Faculty of Agriculture, Mansoura University, El-Mansoura, Egypt

**Keywords:** microbe-mediated nanotechnology, nanobioremediation, plant stress resistance, soil health improvement, sustainable agriculture

## Abstract

Microbial nanotechnology provides a simple, reliable, and eco-friendly method for synthesizing various types of nanoparticles (NPs) by utilizing microorganisms, including viruses, fungi, bacteria, and algae, to manufacture and functionalize them. It has extensive applications in various industries, including electronics, healthcare, food production, environmental science, and agriculture. Environmental shifts have greatly influenced global crop production. Abiotic stresses, including heat, UV radiation, salinity, cold, drought, and heavy metals (HMs), adversely affect crop development and yield production. Nanotechnologies are known as powerful tools for crop improvement, aiming to increase crop yield and stress tolerance. Microbially produced NPs can reduce stress-induced impairments and promote plant development in challenging environments. In this context, microbial-mediated NPs act as multifunctional agents that can modulate plant stress responses, improve nutrient availability, and reduce metal toxicity in soil-plant systems. The recent review highlights a comprehensive overview of microbial NPs synthesis, with a particular focus on the mechanisms underlying NPs formation, NPs-HMs interactions, and their role in improving crop resilience. It further highlights recent advances in microbe-mediated nanotechnology for sustainable agriculture and critically discusses current limitations, including scalability, environmental safety, and field-level application challenges. Finally, future perspectives are presented to bridge the gap between laboratory research and practical agricultural implementation.

## Highlights

1. Microbial nanotechnology produces NPs that help plants withstand abiotic stresses.
2. Crops that incorporate NPs are more resilient to abiotic stress, including HM stress.
3. Microbial nanotechnology enhances crop productivity and resilience in challenging conditions.
4. It promotes sustainable agriculture through nanobioremediation to improve soil health.
5. Microbially generated NPs increase crop yield, which is sensitive to stress.

## 1 Introduction

The interconnected primary economic sectors of agriculture and technology are key drivers of stable and secure economic growth, especially in developing nations. Given current trends in global warming, climate change, population growth, and food security concerns, innovative technological solutions combined with sustainable farming practices are essential. Croplands account for only about 12% of the world’s agricultural land, which encompasses approximately 9.3 billion hectares (Shaikh et al., 2024). To achieve high yields, farmers have used agrochemicals, including pesticides, fertilizers, herbicides, and insecticides, for the past few decades. Currently, this arable land receives 3.53 MT of insecticides and 195.38 MT of fertilizers annually (Shaikh et al., 2024). Consequently, there is a noticeable decline in agricultural health and soil biodiversity, which ultimately impacts human health (Wall et al., 2015). Achieving plant stress tolerance and adaptive behaviors requires technologies beyond traditional breeding; sustainable technologies are essential. Notably, abiotic stresses [including drought, salinity, heat, UV radiation, and especially heavy metal (HM) contamination] further intensify crop yield losses by disrupting plant physiological, biochemical, and molecular processes, thereby demanding innovative and sustainable mitigation strategies. In this regard, nanotechnology has developed as a promising approach to address these agricultural and environmental challenges.

**FIGURE 1:**
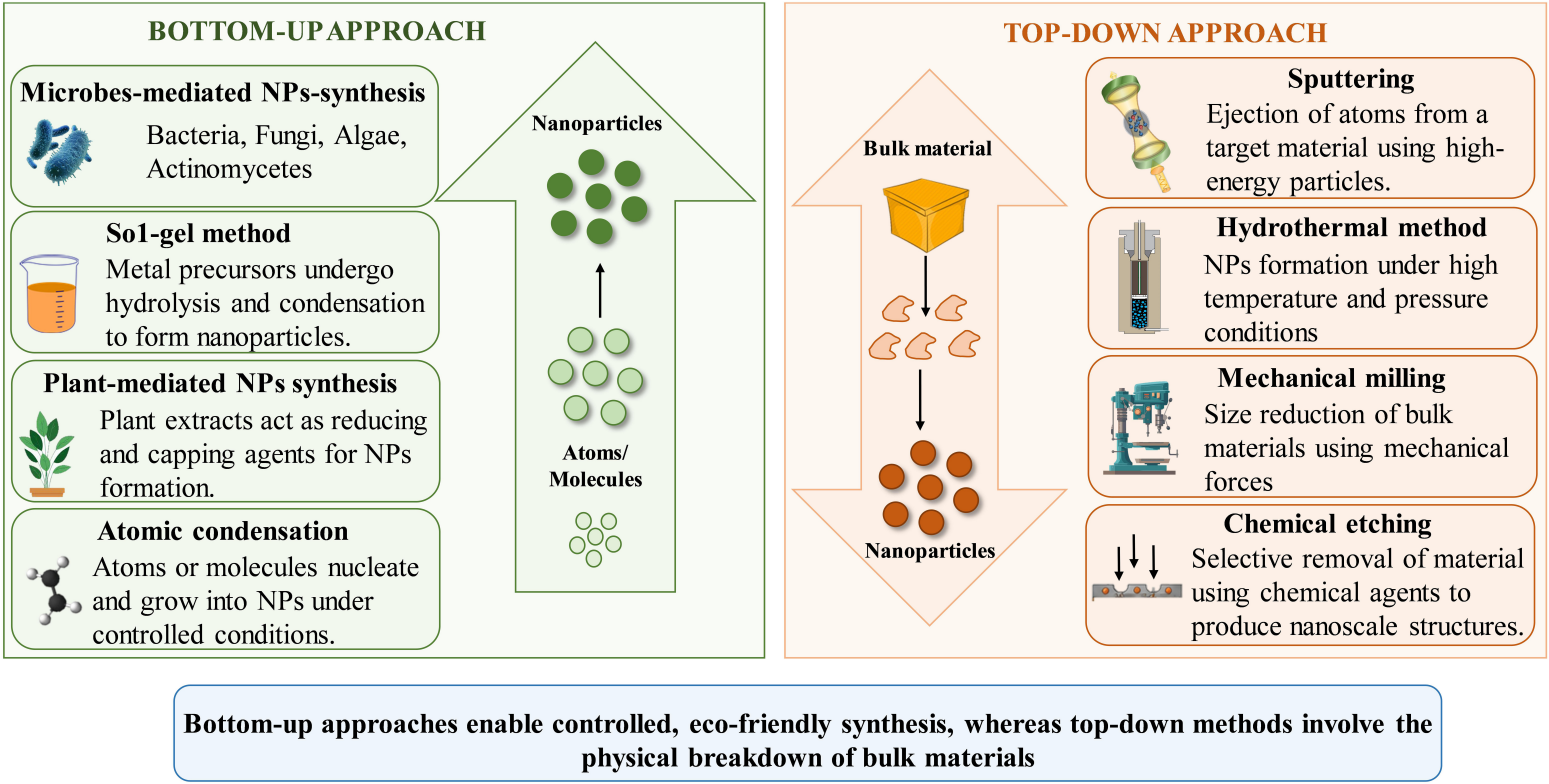
Various approaches to synthesizing nanoparticles.

The 21st century has introduced a new agricultural technique, nanotechnology, first proposed by Richard Feynman in 1960. The study used the phrase “There’s Plenty of Room at the Bottom” when discussing the limitations of various technologies and how they could be used to benefit humankind. Since then, experts have become interested in this evolving technology and have begun applying it in interdisciplinary research projects worldwide. Nanotechnology is defined as “a science, engineering, and technology conducted at the nanoscale (1–100 nm).” Nanoparticles (NPs) are unique due to their physicochemical properties, including their small size, high reactivity, high surface-to-volume ratio, and altered bonding patterns. These characteristics of NPs have enabled them to increase crop yield and stress tolerance in agricultural systems under harsh environmental conditions (Table 1). Currently, two main methods, the top-down and bottom-up approaches, are used to produce NPs (Figure 1), depending on quality, speed, and cost (Ansari et al., 2024; Samaan et al., 2026).

**TABLE 1:** Classification of nanoparticles (NPs) and their functional roles in plant stress regulation.

Category	NP type	Size range	Examples	Functions in plants	Mechanism in plant stress mitigation	References
Organic NPs	Chitosan-based polymers	≤ 100nm	Chitosan nanoparticles (ChNPs)	Enhance plant immunity boost PAL, POX, PPO, protease inhibitors; improve growth and mineral uptake in finger millet under stress (en.wikipedia.org, pubmed.ncbi.nlm.nih.gov)	Induces systemic resistance; activates defense enzymes (PAL, POX, PPO); improves nutrient uptake and antioxidant metabolism	Sathiyabama and Manikandan, 2021
Carbon-based NPs	Carbon allotropes	1–100nm	Graphene oxide (GO), carbon Q-dots	Increase stress resilience; assist pollutant adsorption and sensing (e.g., GO reprograms Cd-stressed tomato metabolism)	Adsorption of toxic metals; metabolic reprogramming; modulation of photosynthesis and ROS balance	Li et al., 2023; Sharifan and Ma, 2021
Metal NPs	Metal oxides (Fe_3_O_4_)	∼19–40nm	Magnetite NPs (Fe_3_O_4_)	Reduce Pb/Cd uptake by ∼90%, upregulate antioxidants, and mitigate oxidative stress in rice	Immobilization of metals in soil; antioxidant enzyme activation; reduction of Pb/Cd translocation	Singh et al., 2023
Metal oxide NPs	ZnO	< 100nm	Zinc oxide NPs	Alleviate Cd stress in tomato via reduced Cd uptake, improved photosynthesis and metabolic reprogramming	Reduces metal uptake; improves chlorophyll content; regulates nutrient and redox balance	Wang et al., 2023
Metal oxide NPs	Fe_2_O_3_	∼10–50nm	Iron(III) oxide NPs	Limit Cd translocation in rice via iron plaque formation and carrier gene regulation	Formation of iron plaque; regulation of metal transporter genes; reduced metal mobility	Zhou et al., 2023
Magnetic NPs	Fe_3_O_4_	5–100nm	Magnetite NPs	Mitigate Cd stress in radish by enhancing antioxidant enzymes (POD, SOD, CAT)	Activation of antioxidant defense system (SOD, CAT, POD); ROS detoxification and membrane protection	Emamverdian et al., 2025

As NPs have increased food’s nutritional value and shelf life while reducing the use of hazardous chemicals as agricultural inputs, their application in agriculture has drawn attention (Konappa et al., 2024). They have been broadly used as nanosensors, nanopesticides, and nanofertilizers to increase plant production and growth in harsh environments (Mandal and Sarkar, 2024). Nevertheless, concerns regarding hazardous chemicals and the production procedures for chemically synthesized NPs must be addressed. Manufacturers have recently embraced “microbial synthesis,” a green technique that combines bacteria, fungi, algae, and viruses to produce clean, biocompatible, and environmentally friendly NPs that confer stress resistance in crops (Baker et al., 2017; Vyas et al., 2024).

Although recent studies have documented biogenic NPs synthesis, plant stress tolerance, and HMs detoxification separately. Nonetheless, the integrated understanding of microbe-mediated NPs as a unified system remains limited. Most studies focus either on synthesis mechanisms or on plant responses, with less consideration of the integration between microbial production processes, NPs-metal interactions, rhizosphere effects, and plant stress signaling. Therefore, our review offers a more integrated perspective via associating microbial diversity in NPs synthesis, formation, and stabilization mechanisms, NPs-HMs interactions in soil-plant systems, and their roles in plant stress tolerance. Besides, it critically highlights current limitations and the gap between laboratory findings and field-level application.

### 1.1 Literature search strategy

This review was designed based on a comprehensive analysis of recent peer-reviewed literature regarding microbe-mediated NPs synthesis, HMs stress mitigation, plant responses, and sustainable agricultural applications. Therefore, relevant studies were identified through various databases, such as Web of Science, Scopus, PubMed, and Google Scholar, using keywords such as microbial nanoparticles, biogenic nanoparticle synthesis, heavy metal stress, plant stress tolerance, nanobioremediation, and plant-microbe interactions. The recent studies were prioritized. However, the earlier studies were added when they were required to elaborate on fundamental mechanisms. The studies were selected based on their relevance to microbial NPs production, NPs-mediated detoxification mechanisms, and agricultural applications.

## 2 HMs contamination and its impacts on plants and environmental health

**FIGURE 2:**
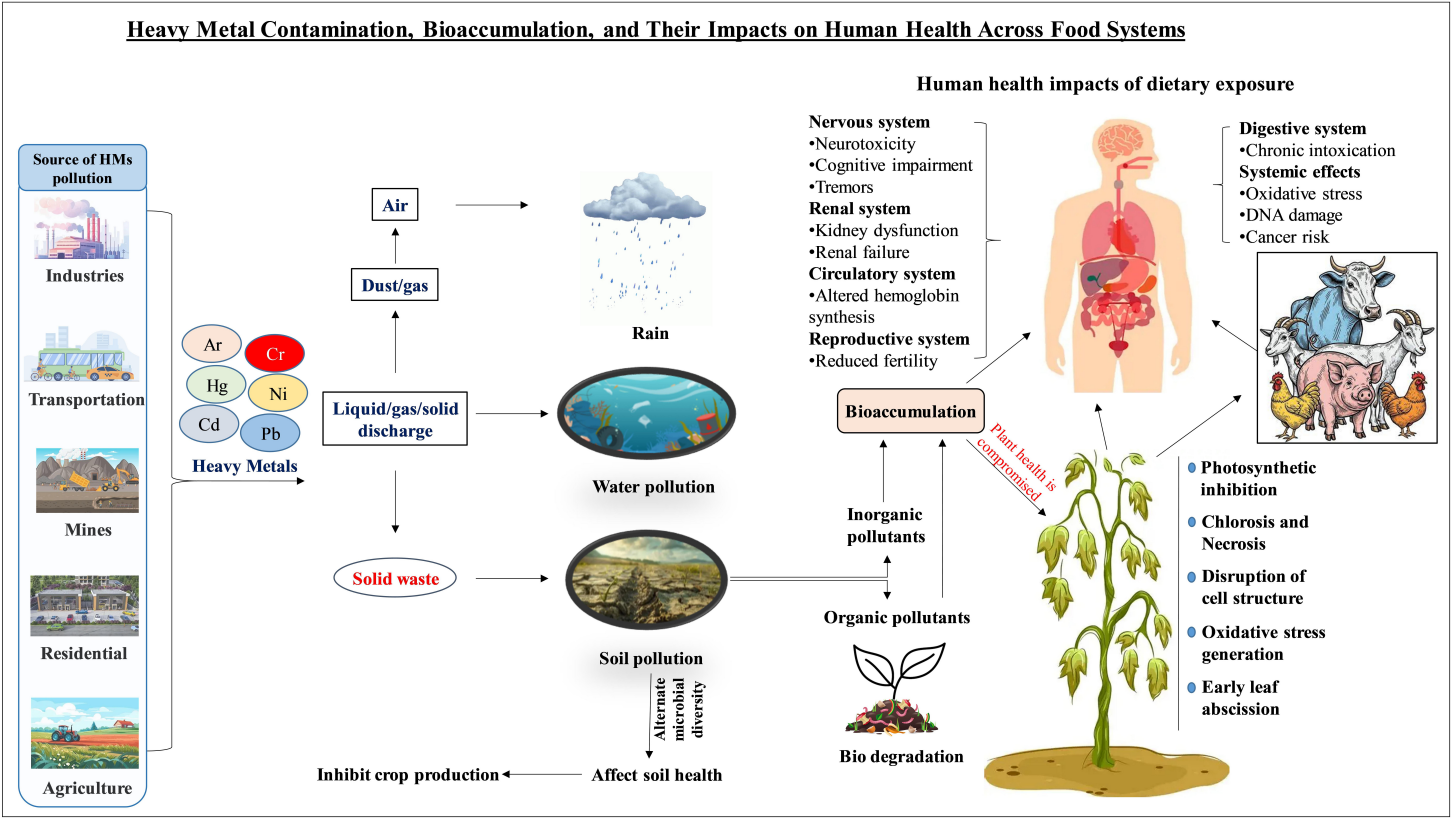
Heavy metals (HMs)’ toxicity to plants, soil, and the environment. The harmful impacts of HMs on soil fertility, plant physiology, and general environmental health are depicted in the image. The image illustrates specific toxicity pathways, including soil degradation, oxidative stress, and disruption of plant enzyme processes.

The primary source of HMs is human activity, which has disrupted soil fertility, productivity, and microbial ecosystems (Sharma and Saraf, 2023; Badamasi et al., 2026). Their accumulation in soil and plants poses serious risks to ecosystems because they bioaccumulate and biomagnify along the food chain (Alengebawy et al., 2021). The permanence, high toxicity, and resistance of metals have made them a major global concern for plants and the environment. These toxic metals pose a serious threat to environmental stability and the health of all living organisms (Tang et al., 2024). High concentrations of HMs in soil are absorbed and accumulated by plants, then enter the food chain, ultimately impacting human nutrition (Sharafi and Salehi, 2025; Figure 2). Like other living organisms, plants are susceptible to elevated concentrations of HMs resulting from human activities and environmental factors. HMs tend to accumulate in plants, disrupting their biological and metabolic functions and ultimately leading to significant yield losses. For instance, HMs inhibit seed germination, induce elevated levels of reactive oxygen species (ROS), e.g., hydrogen peroxide (H_2_O_2_) and malondialdehyde (MDA), adversely affect plant water status, weaken cell membranes, and intensify the loss of vital osmolytes (Hasanuzzaman et al., 2021). Various chemical, physical, and biological techniques are used globally to remove HMs from soils. The complexity of HM interactions with soil-plant systems makes conventional remediation approaches insufficient under field conditions. Maintaining soil quality, which is essential for sustainable agriculture, depends heavily on soil biology. Soils contaminated with HMs are treated biologically using plants and microbes (Khalid et al., 2017).

## 3 Microbial-assisted HM phytoremediation

Recent research suggests that microbial bioremediation has significant potential for cleaning contaminated soils. Most HMs are classified as harmful, and a promising green technology approach involves harnessing the metabolic processes of microorganisms to eliminate HMs contamination. Scientists worldwide are increasingly focused on microorganisms for their potential in several bioremediation methods, including biosorption, bioaccumulation, biovolatilization, biomineralization, oxidation and reduction, bioleaching, and biosurfactant production. These microbes absorb and accumulate HMs from soil at high concentrations, thereby mitigating contamination (Zou et al., 2025). Due to their small size, microbes offer a large surface area for adsorbing HMs, thereby decreasing the overall HMs levels (Rajput et al., 2022).

Microorganisms can reduce HM contamination in soil; for instance, *Stenotrophomonas rhizophila* reduced Pb and Cu by 76.9 and 83.4%, respectively (Sun et al., 2021), while *Aspergillus niger* showed a remarkable ability to bioaccumulate Cd and Cr (Rajput et al., 2022). Still, the regulatory interactions between microbial communities and plant signaling pathways (e.g., hormone modulation and gene expression responses to HM stress) remain poorly understood, which limits the predictive ability of current phytoremediation models.

## 4 Interactions between NPs and HMs in plant detoxification

The presence of HMs and NPs in agronomic areas can have additive or synergistic harmful effects on crop yields, soil structure, and microbiome dynamics (Basit et al., 2022a). Though, a variety of biological and environmental factors affect the interaction dynamics between HMs and NPs (Basit et al., 2022b; Basit et al., 2023a). Furthermore, biotic factors interfere with the NPs’ uptake, transport, and accumulation across plant organs. The interaction of HMs in contaminated environments is primarily mediated by chemical interactions, physical adsorption, and electrostatic binding (Bilal et al., 2021; Table 2). For instance, owing to their unique characteristics, including reactivity, electrostatic attraction, large surface area, and capping molecules, FeO-NPs can adsorb Cd from soil effectively (Manzoor et al., 2021). These interactions are further impacted at the plant-soil interface by ion displacement, surface complexation, and transporter competition, which together demonstrate metal bioavailability.

**TABLE 2:** Mechanistic interactions between nanoparticles (NPs) and heavy metals (HMs) in plant stress mitigation.

Nanoparticles type	Heavy metal	Plant species	NPs-mediated effects	Primary detoxification mechanism	References
TiO_2_ NPs	Cd	Wheat (*Triticum aestivum*)	Reduced Cd toxicity; enhanced growth	Enhanced antioxidant enzyme activity; reduced Cd uptake and oxidative stress	Younas et al., 2025
ZnO NPs	Pb	Rice (*Oryza sativa*)	Improved photosynthesis; reduced Pb accumulation in shoots	Reduced Pb translocation to shoots; improved photosynthetic pigments	Akhtar et al., 2021
Fe_3_O_4_ NPs	Cr	Maize (*Zea mays*)	Reduced Cr accumulation in tissues	Adsorbed Cr in soil; reduced Cr accumulation in roots and shoots	Yousaf et al., 2024
SiO_2_ NPs	Hg	Soybean (*Glycine max*)	Enhanced seedling growth under Hg stress	Improved seedling growth; modulated metal-induced oxidative stress	Li et al., 2020
CeO_2_ NPs, Fe_3_O_4_, SnO_2_, and TiO_2_	Ag, Co, Ni	Tomato (*Solanum lycopersicum*)	Reduced toxicity and improved antioxidant defense	Reduced arsenic bioavailability; enhanced antioxidant defense system	Carbone et al., 2014
Carbon-based NPs	Cd, As	Rice (*Oryza sativa*)	Reduced metal uptake and phytotoxicity	Immobilized Cd and As in the root zone; limited uptake and toxicity	Ma et al., 2021

In this context, Cd transport into plant cells is facilitated by Ca and Fe transporters. Ionic radius similarity and transporter affinity are the main drivers of this competition, where metals bound to NPs decrease the availability of free ionic Cd^2+^ in the rhizosphere. Consequently, fierce competition occurs between Cd and FeO-NPs for entry into plant systems via the same transporter channel, which lowers the metal content in plant tissues (Ahmed et al., 2021). Likewise, Noman et al. (2020) indicated that Cu-NPs’ electrostatic attraction, reactivity, and large surface area restricted Cd transportation from soil systems to upper wheat organs. Therefore, the capping molecules of biogenic Cu-NPs enhanced the soil’s ability to immobilize Cd. Thus, wheat growth was aided by the absorption of Cu-bound nutrients (Noman et al., 2020).

Moreover, it activates enzymes involved in plant development in contaminated soils and functions as a coenzyme in other important reactions. Excessive NPs accumulation may also alter beneficial soil microbial activity, requiring careful dosage optimization. Similarly, HMs ions have been extracted from contaminated areas using graphene oxide (GO), which has a very large surface area (Etemadi et al., 2017; Raidongia et al., 2014). For instance, GO sheets that may conjugate with HMs, such as Cr (VI), due to their functional groups, can optimize the kinetics of HMs ion absorption (Etemadi et al., 2017; Raidongia et al., 2014). The pH, ionic strength, and organic matter content of the surrounding environment have a substantial impact on the effectiveness of these interactions and can drastically alter adsorption capacity under field conditions. In conclusion, the ability of NPs to form metal complexes is likely a key factor in understanding NPs-mediated metal stress alleviation and offers a promising strategy for eliminating the detrimental effects of HMs in various environmental and biological systems (Wang L. et al., 2017; Wang X. et al., 2017). Despite promising laboratory outcomes, variability in soil composition and NPs stability remains a major constraint for consistent field performance.

## 5 Microbiome-HMs interactions

There is a dynamic interaction between the soil microbiome and HMs that plays a key role in bioremediation. Microorganisms can directly influence the form and activity of HMs in different environments. During the metal mobilization phase, microorganisms can affect the composition and amount of root secretions. For example, a review of the enrichment mechanisms of As, Cd, Pb, and Zn indicates that rhizosphere microorganisms, such as *Bacillus subtilis*, promote metal chelation and precipitation by regulating plant discharge of organic acids with low molecular weight, thereby influencing the solubility and bioavailability of HMs (Asare et al., 2024). Furthermore, microorganisms can secrete low-molecular-weight organic iron carriers that specifically chelate Fe^3 +^. Research on soil microorganisms in agriculture indicates that strains such as *Sinorhizobium meliloti*, *Variovorax paradoxus*, and *Pseudomonas fluorescens* can secrete siderophores and ACC deaminase. For example, *V. paradoxus* can synthesize siderophores, thereby enhancing nickel absorption and uptake by plants and reducing nickel toxicity (Khatoon et al., 2024). During the metal immobilization stage, biofilm membrane structures composed of extracellular polymers produced by microorganisms on root surfaces act as a “protective shield,” adsorbing metal ions and preventing HMs from entering plant roots. Another study shows that *Klebsiella variicola* and *Pseudomonas otitidis* from mining areas can form biofilms in the rhizosphere of mustard greens, enhancing microbial colonization and metal stability. These biofilms facilitate controlled metal transfer and plant uptake by increasing the solubility of Ni and Pb (by 13.3- and 10.7-fold, respectively), thereby improving phytoremediation efficiency and preventing metals from entering the plant in highly toxic forms (Sharma and Saraf, 2023).

Additionally, extracellular polymeric substances (EPS), composed of macromolecules, i.e., sugars, proteins, lipids, and nucleic acids, can effectively adsorb HMs’ ions. In 2024, Zhu et al. (2024) designed engineered *B. subtilis* biofilm@biochar materials for onsite detection and remediation of Pb^2+^, Cu^2+^, and Hg^2+^ in the environment. This system adsorbs these metals via EPS, reducing metal accumulation in plant roots (Zhu et al., 2024). Finally, in the metal-redox transformation state, microorganisms that produce ACC deaminase lessen plant ethylene levels against metal stress, thereby alleviating phytotoxicity and enhancing plant tolerance through improved physiological regulation and redox balance. For instance, Mohamed et al. (2025) found that rhizospheric actinomycetes producing ACC deaminase under HMs stress significantly increased the height, dry weight, and chlorophyll content of maize seedlings in Pb (1,000mg kg^–1^) contaminated soil.

In the rhizosphere under HMs toxicity, diverse microbial communities synergistically evolved mechanisms that markedly enhanced plant adaptability and promoted ecosystem recovery. Rhizospheric bacteria (PGPR) increase metal solubility by regulating root secretions, synthesizing iron carriers, and secreting ACC deaminase, thereby enhancing metal uptake, metal tolerance, and plant growth (Shaffique et al., 2023). Fungal communities expand the root absorption area via hyphal networks and form biofilms and EPS in the rhizosphere to adsorb or passivate HMs, enhancing plant tolerance to metal stress. Endophytes that share mechanisms such as ACC deaminase and siderophores can directly participate in metal detoxification and transformation through chelation, precipitation, and enzymatic sulfhydrylation, while promoting plant antioxidant systems and gene regulation (Bhardwaj et al., 2025). Meanwhile, HMs are highly toxic to microorganisms, particularly at elevated concentrations or when exposed to highly toxic metals such as cadmium. These conditions can disrupt microbial metabolism and reduce their ability to perform beneficial processes such as bioremediation. Even low levels of cadmium (around 2.5mg kg^–1^) can substantially inhibit soil microbial activity, community diversity, biomass, and enzymatic functions (You et al., 2024). The toxicity mechanisms include excessive ROS production, which causes oxidative stress in microbial cells (Mansoor et al., 2023); inducing DNA and protein damage leading to genotoxic stress (Rath and Das, 2023); disrupting cell membrane integrity and altering permeability, leading to uncontrolled ion leakage and homeostasis imbalance (Basit et al., 2024); and, with prolonged exposure, reducing activities of key antioxidant enzymes like superoxide dismutase (SOD) and catalase (CAT) (You et al., 2024). Mercury can inhibit transcription, arsenic can cause DNA damage, and other metals, such as copper, nickel, and zinc, can decrease enzyme activity and induce cellular disorders (Pant and Singh, 2024; Zhang et al., 2023). Despite this toxicity, many microorganisms have evolved resistance mechanisms through long-term adaptation, enabling them to survive in contaminated soils. HMs-tolerant microbes can adapt by evolving detoxification systems and stress-responsive genes, enabling survival and function in stressed environments (Li et al., 2024). Recent studies illustrated that metal-resistant rhizobacteria can transform toxic HMs into less bioavailable or non-toxic forms through redox reactions, complexation, volatilization, and precipitation (Fu et al., 2024). These microbial communities, which promote plant growth, are becoming vital resources for ecological restoration and soil remediation. Briefly, microorganisms are not only victims of HMs pollution but also key “ecological engineers actively involved in regulation and repair mechanisms. By influencing metal mobility, availability, and toxicity, they improve plants and soil health, forming a complex “microbe-metal-plant” ecological system.

## 6 Microbe-mediated synthesis of NPs

**FIGURE 3:**
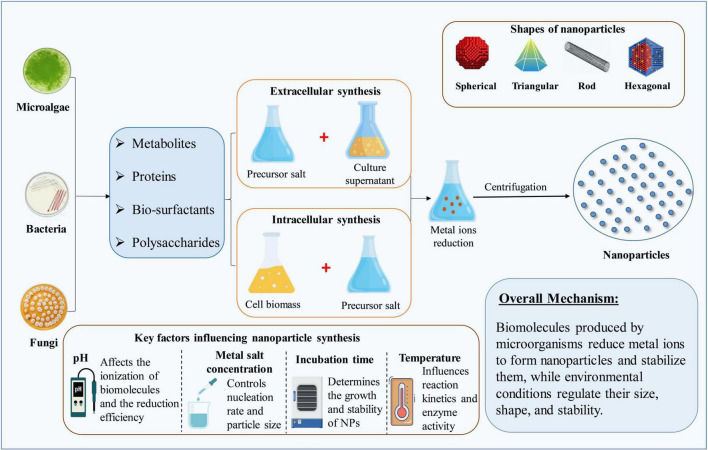
Microorganisms-mediated NPs generation through the reduction of metal ions they encounter. These metal ions are transformed into nanoparticles, which are stabilized by biomolecules, such as enzymes and proteins, produced by the microbes. The characteristics of NPs are affected by the microorganism, processing conditions, and the available metal ions.

Conventional chemical and physical methods exist for synthesizing NPs, including vapor condensation, solvent evaporation, physical fragmentation, interferometric lithography, microemulsion precipitation, and the sol-gel method (Agarwal et al., 2018). These procedures often involve hazardous materials that contribute to environmental pollution. Additionally, these toxic compounds can accumulate in plants, posing risks to human health as NPs become part of the food chain through consumption (Isibor et al., 2024; Nechitailo et al., 2025). In contrast, microbe-mediated NPs production has various advantages over traditional chemical techniques. This method is biocompatible, economical, and environmentally friendly (Shinde et al., 2023). Microorganisms (such as bacteria, fungi, yeast, and microalgae) can naturally produce highly stable NPs, making them an appropriate option for NPs formation (Kuppusamy et al., 2025; Figure 3). Furthermore, the size, shape, and composition of NPs can be precisely controlled through microbial production, thereby significantly affecting their characteristics and potential applications (Kirubakaran et al., 2025). Research has highlighted that the efficacy of biogenic NPs largely depends on their particle size, dispersion, and stability (Bokolia et al., 2025). Many studies show that microbial NPs formation techniques can be used to regulate and manipulate their size. In addition, modifications to the surfaces of microbially produced NPs can enhance their dispersion, thereby improving interactions with targeted pathogens (Swetha et al., 2025). The stability of these NPs, which is influenced by the capping agents microbes elicit, is also beneficial for their persistent antibacterial effectiveness (Osonga et al., 2020).

Mechanistically, microbial biomolecules such as proteins, polysaccharides, and secondary metabolites support the nucleation and growth processes that follow enzymatic reduction of metal ions. These biomolecules directly affect the stability, shape, and functional performance of NPs by acting as natural capping and reducing agents.

### 6.1 Microalgae-based biological NPs production

Numerous benefits accompany the biosynthesis of NPs using microalgae, including low costs, high production rates, and ease of proliferation (Chan et al., 2022). Harvesting microalgae is straightforward, enabling efficient processing to produce NPs. This method is also environmentally friendly, as it utilizes fewer hazardous chemicals and generates less waste (Dahoumane et al., 2017). Microalgae, photosynthetic microorganisms, can convert metal ions into NPs via internal or extracellular enzymes. Numerous studies have assessed the extracellular and intracellular biosynthesis approaches used to produce biogenic NPs from microalgae (Do et al., 2025). Enzyme-mediated redox reactions mainly drive biosynthetic processes and photosynthetically produced reducing equivalents, which promote metal ion reduction and NPs synthesis.

For example, da Silva Ferreira et al. (2017) produced silver chloride NPs using the microalgal species *Chlorella vulgaris* and assessed their antibacterial activity against harmful bacteria. Similarly, Çalışkan et al. (2019) showed that the microalga *Galdieria* sp. mediated the production of zinc, iron, and silver NPs. They employed conventional material characterization techniques, such as UV-Vis spectroscopy and Fourier transform infrared spectroscopy (FTIR), to characterize these NPs (Çalışkan et al., 2019). Likewise, green-synthesized Cu NPs by the microalgae *Dunaliella tertiolecta*, *Tetraselmis suecica*, and *Chlorella kessleri* have been reported in several studies (Salas-Herrera et al., 2019). Nonetheless, variances in metabolic activity, species-specific enzymatic profiles, and culture conditions have a substantial impact on NPs yield and uniformity, thereby limiting reproducibility at large scale. In brief, microalgae-mediated NPs synthesis represents a viable and environmentally friendly method. However, further investigation is needed to optimize the synthesis process and to elucidate the fundamental mechanisms underlying the production of microalgae-based NPs.

### 6.2 Biosynthesis of NPs using bacterial strains

NPs produced by bacteria are a sustainable, environmentally friendly method with several agricultural applications (Bano, 2025). Since bacteria can produce extracellular enzymes that convert metal ions into identical NPs, they have been used to synthesize NPs. Bacterial-mediated NPs formation can occur via several mechanisms, including intracellular and extracellular biosynthesis, and bioaccumulation (Shah et al., 2025). During bacterial-mediated NPs synthesis, redox-active enzymes such as reductases and oxidoreductases play a central role in converting metal ions into zero-valent NPs. Meanwhile, extracellular polymeric substances (EPS) contribute to stabilization and avoid aggregation.

Nevertheless, intracellular biosynthesis is the mechanism by which bacteria formulate NPs within their cells. During this procedure, bacterial cells absorb metal ions, and intracellular enzymes reduce them to form NPs. The NPs are released into the surrounding environment upon bacterial cell lysis. On the other hand, extracellular biosynthesis is the process by which bacteria generate NPs outside their cells (Bokolia et al., 2025). Through this process, extracellular enzymes are secreted, enabling the conversion of metal particles into the appropriate NPs. NPs can be extracted and purified from the extracellular milieu following their release (Spagnoletti et al., 2019).

Several investigations illustrated the sustainable production of metallic NPs using bacteria (Bokolia et al., 2025; Kılıç, 2025). Ahmed et al. (2020) reported the biosynthesis of silver NPs using *B. cereus* to suppress bacterial diseases in rice. Meanwhile, Varshney et al. (2010) verified the efficient production of copper NPs by using *Pseudomonas stutzeri*, which was obtained from wastewater. Despite these advantages, bacterial-mediated NPs formation is highly sensitive to environmental parameters such as pH, temperature, and nutrient availability, which can considerably affect NPs uniformity and production. Additionally, several bacterial strains have been utilized for the production of microbe-mediated NPs, including *B. amyloliquefaciens*, *Escherichia coli*, *Acinetobacter calcoaceticus*, *P. stutzeri*, and *Lactobacillus* sp. (Husseiny et al., 2007; Khan and Fulekar, 2016; Selvarajan and Mohanasrinivasan, 2013; Singh et al., 2013). However, reductase is an essential enzyme in the green synthesis of copper, silver, and zinc NPs from *Streptomyces* sp. (Karthik et al., 2014). Overall, bacterial-mediated NPs biosynthesis is a practical and sustainable method for NPs production.

### 6.3 NPs biosynthesis via fungi

Fungi are a diverse group of organisms known for their ability to produce an array of extracellular enzymes and metabolites, and have been extensively utilized for the synthesis of NPs. Fungal-mediated NPs production is relatively efficient due to their high secretion capacity of enzymes and biomolecules, which boost metal ion reduction and NPs stabilization. These substances released by fungi can effectively reduce metal ions to the desired NPs. Various methods, including both internal and extracellular biosynthesis and the use of fungal biomass, can be employed to produce NPs from fungi (Meera et al., 2025). Fungal cells absorb metal ions during intracellular biosynthesis, where they can be transformed into NPs by various intracellular enzymes. After cell lysis, these NPs are released into the extracellular environment (Tyagi et al., 2019). Extracellular enzymes that may convert metallic ions into their equivalent NPs are released during extracellular biosynthesis (Zhang et al., 2019). Plenty of studies have highlighted the advantages of fungal-based NPs synthesis, such as high production rates, low cost, and ease of cultivation (Al-Goul, 2025; Tomah et al., 2020). Simultaneously, fungal-mediated NPs synthesis may face challenges such as long cultivation time, contamination risk, and difficulty in controlling NPs size distribution.

For instance, Tomah et al. (2020) confirmed the generation of silver NPs using *Trichoderma virens* HZA14, the cell-free filtrate, which was efficient at eradicating *Sclerotinia sclerotiorum* infection (Tomah et al., 2020). Also, *A. niger* has been reported to produce zinc oxide NPs that shielded potato (*Solanum tuberosum* L.) plants from *Alternaria solani*, a pathogen that causes early blight disease (Singh et al., 2022). Likewise, Jain et al. (2013) described the production of zinc oxide NPs mediated by *A. aeneus*. Similarly, yeasts have also been used for large-scale biosynthesis of NPs (Meera et al., 2025; Rai et al., 2025). Hence, fungal-mediated NPs formation offers a flexible, cost-effective, and sustainable approach for environmentally friendly production of NPs that possess unique qualities and potential applications in agriculture. Nevertheless, additional studies in this field may result in the development of novel, eco-friendly practices and technologies.

## 7 Nanobioremediation using microbial NPs

**FIGURE 4:**
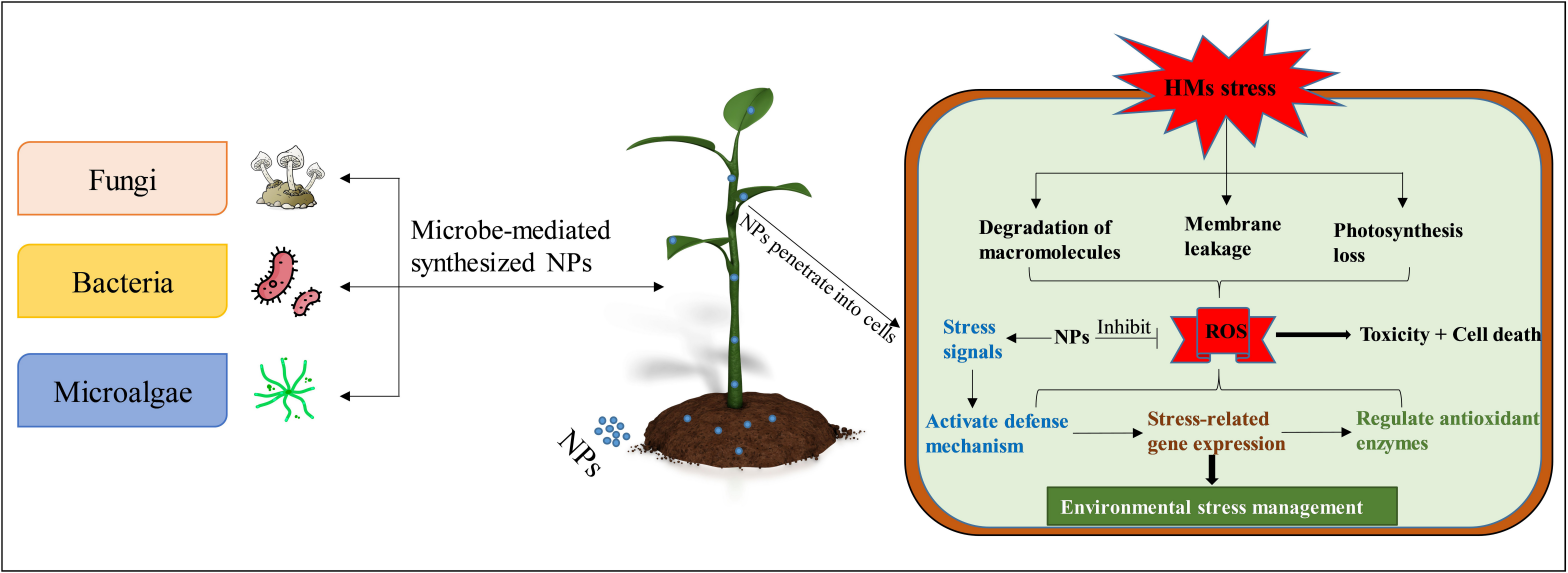
The use of microbes-mediated synthesized NPs to strengthen plants’ defenses against abiotic stress.

Rapid globalization drastically contaminates the environment by accelerating the release of hazardous metals. When these HMs accumulate in the soil, they adversely affect soil dynamics and disrupt microbial structure, impair nutrient cycling, resulting in reduced soil fertility and lower crop productivity (Shaikh et al., 2025). The stress caused by HMs adversely impacts photosynthetic efficiency, water potential, and overall growth characteristics of crops, frequently resulting in crop damage (Basit et al., 2023b). In this regard, microbe-mediated NPs have become versatile agents that control stress signaling pathways and plant antioxidant defense systems alongside immobilizing HMs. Although, using microorganisms to generate NPs is an interesting approach for developing nanomaterials that improve plants’ ability to withstand HMs stress (Figure 4; Khan et al., 2025). For instance, Venkatachalam et al. (2017) examined the effects of zinc oxide NPs (ZnONPs) at 25 mg L^–1^ on *Leucaena leucocephala*. These NPs were produced using *Ulva lactuca* in response to HMs stress, comprising lead (Pb) at 100 mg L^–1^ and cadmium (Cd) at 50 mg L^–1^. Green-synthesized ZnONPs substantially enhanced plant growth and increased photosynthetic pigment levels in HMs-stressed *L. leucocephala* (Venkatachalam et al., 2017). Reduced metal bioavailability and increased ROS scavenging through enzymatic antioxidant activation are the main contributors to this improvement. A comparative synthesis of representative studies on NPs-mediated alleviation of HMs stress in plants is presented in Table 3.

**TABLE 3:** Comparative overview of chemical remediation, phytoremediation, and microbe-mediated NPs systems for HMs-contaminated soils based on cost, efficiency, scalability, and environmental risks reported in earlier studies (Ali et al., 2013; Alloway, 2013; Badamasi et al., 2026; Olawade et al., 2024).

Approach	Cost	Efficiency	Scalability	Environmental risk	Limitations
Chemical remediation (e.g., soil washing, chelation, immobilization agents)	High (reagents, infrastructure, labor-intensive)	High short-term metal removal efficiency	Moderate to low (site-specific engineering required)	High (soil structure disruption, secondary pollution, nutrient loss)	Expensive, non-selective soil fertility degradation
Plant-based phytoremediation	Low to moderate	Moderate (slow uptake-dependent process)	High (field deployable over large areas)	Low (eco-friendly, minimal disturbance)	Time-consuming, limited to bioavailable metals, and seasonal dependency
Microbe-mediated NPs system	Moderate (emerging, production cost decreasing)	High potential (adsorption + detoxification + plant signaling modulation)	Moderate (currently limited by scale-up and formulation stability)	Uncertain (potential NPs accumulation and microbial imbalance)	Field instability, regulatory gaps, and ecotoxicity uncertainty

Similarly, treatment with iron oxide (Fe_3_O_4_) NPs at 0.5 mg g^–1^, synthesized from the husk extract of *Cocos nucifera*, improved plant biomass, the quantum efficiency of photosystem II (PSII), and chlorophyll content in cadmium-stressed *Oryza sativa*, resulting in increased crop productivity (Sebastian et al., 2018). In addition, Sebastian et al. (2019) reported that Fe_3_O_4_ NPs (0.5 g) generated via the bark extract of *Hevea brasiliensis* significantly augmented plant biomass, enhanced photosynthetic pigments, and upheld nutrient homeostasis, reduced the accumulation of Cd^2+^ ions, and minimized oxidative damage prompted by stress in *O. sativa*. The observed effects are associated with NPs-mediated metal adsorption in soil and with limited translocation of toxic ions from roots to shoots.

In another study, the foliar supplementation of TiO_2_ NPs (100 mg L–1), generated from leaf extracts of *Trianthema portulacastrum* and *Chenopodium quinoa*, significantly repressed Cd^2+^ absorption in the plant system. This treatment also improved plant height, spike length, chlorophyll content, and grain yield in *Triticum aestivum* (Irshad et al., 2021). This reduction in Cd^2+^ uptake is predominantly due to altered membrane transport activity and reduced metal mobility in plant tissues. Correspondingly, employing SNPs (100 μM) derived from *O. basilicum* leaf extract increases *Helianthus annuus*’s resistance to stress-mediated responses imposed by manganese (Mn) (Saad-Allah and Ragab, 2020). This treatment upsurges the levels of crude protein, total amino acids, and cysteine, while reducing ROS-mediated lipid peroxidation. Also, the exogenously applied TiO_2_ NPs (0.1%) derived from the leaf extract of *Musa paradisiaca* significantly reduce As^3+^ accumulation in *Vigna radiata* under arsenic (As) stress (Katiyar et al., 2020). This treatment also improves protein content and boosts enzymatic antioxidant activities, including SOD, ascorbate peroxidase (APX), and CAT, thereby promoting ROS detoxification (Venkatachalam et al., 2017).

Overall, these investigations indicate three primary mechanisms for NPs-mediated HMs detoxification: (i) immobilization and adsorption of metal ions in soil, (ii) reduced uptake and translocation of toxic ions in plant tissues, and (iii) activation of antioxidant defense systems that lower oxidative stress, thereby enhancing plant resilience under HMs stress.

## 8 Conclusion and prospects

HMs contamination is a substantial global problem and is regarded as one of the greatest challenges of this century. The impact of HMs pollution on soil fertility, quality, microbiological activity, and biodiversity is distressing. It can also enter the food chain, adversely affecting human health. Various physicochemical techniques are used worldwide to remediate HMs-contaminated soils; while, these methods can alter soil quality, are often expensive, difficult to implement, and may be ineffective in certain cases. Consequently, the development of new biological approaches to remediate contaminated soils is crucial. As a useful alternative, HMs-induced plant stress could be mitigated by applying nanotechnology, particularly in the microbial-mediated NPs formation. NPs’ potential for tailored administration and increased efficacy has made them a novel and successful strategy for mitigating plant stress. Some drawbacks of conventional chemical pesticides, including limited specificity and adverse environmental effects, may be mitigated by using microorganisms to produce NPs. Microbial-based NPs have the potential to reduce environmental contamination by targeting HMs-induced stress tolerance and decreasing the HMs uptake and accumulation. Despite this, several important limitations and knowledge gaps must be addressed to ensure the secure use and societal acceptance of NPs in the environment. Key issues include optimizing NPs production, maintaining their stability and bioavailability, and facilitating efficient delivery to the intended sites. It is also crucial to address concerns regarding the safety and ecological impacts of NPs. Notably, climatic variability, NPs aggregation, and uneven performance across various soil systems hinder the application of microbial-NPs technologies to field conditions despite robust laboratory and greenhouse-scale results.

To maximize the potential of biological synthesis techniques on an industrial scale, it is essential to focus on benefits such as environmental friendliness, scalability, and cost-efficacy. Considering the potential uses of NPs in agriculture, there is a need to develop and commercialize new tools for the smart management of HM detoxification. One potential area of research is the incorporation of these NPs with advanced technologies, such as gene editing and precision agriculture. Precision agriculture provides real-time data on plant health and stress management, helping farmers efficiently apply appropriate remedial measures, often using NPs. However, both farmers and researchers often lack a clear understanding of the optimal application methods for these NPs and of how to assess their effectiveness. As well, a regulatory framework is needed to ensure the safety, consistency, and effectiveness of these NPs. In addition, comprehensive risk assessment frameworks are mandatory to assess the long-term environmental fate, ecotoxicological impacts, and potential accumulation of NPs in soil-plant systems before large-scale deployment.

To effectively manage the efficacy, stability, repeatability, and impact of microbe-mediated NPs in real agricultural environments, conducting field trials is essential. Nanoscale NPs must not adversely affect environmental processes, beneficial microbial ecosystems, or plant growth. Further research is needed to overcome these obstacles and fully utilize the potential of microbe-mediated NPs. We believe that our review will contribute to the development of innovative NPs production and application systems that efficiently and cost-effectively alleviate HMs stress in plants, thereby supporting global food security and enhancing sustainable agricultural systems.

Concisely, this review offers a deeper understanding by connecting microbial NPs formation, HM detoxification pathways, and plant stress physiology as compared to earlier reported studies.
